# Correction: Analysis of low resource setting referral pathways to improve coordination and evidence-based services for maternal and child health in Ethiopia

**DOI:** 10.1371/journal.pone.0316559

**Published:** 2024-12-26

**Authors:** The PLOS ONE Staff

There are errors in the Author Contributions. The correct contributions are:

**Conceptualization:** Geletaw Sahle Tegenaw, Demisew Amenu, Girum Ketema, Frank Verbeke, Jan Cornelis, Bart Jansen.

**Data curation:** Geletaw Sahle Tegenaw.

**Formal analysis:** Geletaw Sahle Tegenaw.

**Investigation:** Geletaw Sahle Tegenaw.

**Methodology:** Geletaw Sahle Tegenaw, Demisew Amenu, Girum Ketema, Frank Verbeke, Jan Cornelis, Bart Jansen.

**Software:** Geletaw Sahle Tegenaw.

**Supervision:** Demisew Amenu, Girum Ketema, Frank Verbeke, Jan Cornelis, Bart Jansen.

**Validation:** Geletaw Sahle Tegenaw, Demisew Amenu, Girum Ketema, Frank Verbeke, Jan Cornelis, Bart Jansen.

**Visualization:** Geletaw Sahle Tegenaw, Frank Verbeke, Jan Cornelis, Bart Jansen.

**Writing–original draft:** Geletaw Sahle Tegenaw.

**Writing–review & editing:** Geletaw Sahle Tegenaw, Demisew Amenu, Frank Verbeke, Jan Cornelis, Bart Jansen.

The publisher apologizes for the error.
