# Supplementary material for: Analysis of low resource setting referral pathways to improve coordination and evidence-based services for maternal and child health in Ethiopia
Source: PLoS One. 2022 Aug 25;17(8):e0273436. doi: 10.1371/journal.pone.0273436 (PMC9409580; doi:10.1371/journal.pone.0273436)
Supplement: Attachment — Submitted filename: REVIEWERS COMMENTS_Plos One.docx [file pone.0273436.s001.docx]

**REVIEWERS COMMENTS**

**ABSTRACT**
In the Abstract section the results indicate that out of 201. It is not clear what the N(number of matching samples are that lead to the 13%). Kindly indicate this. The results of the study are not compatible with the objectives. The objectives indicate that the study intended to explore the practice whereas the results indicate the main reasons for referral. Kindly align the two. The conclusion section should indicate the overall implication of the study within the health system. It currently reads like an objective. Kindly revise to indicate the implication.

**DATA COLLECTION SECTION**

In page 11 you mention that 459 cases were used. It is not clear how the sampling was done. As much as the authors indicate the stepwise approach the calculation that is present in the paragraph below does not match the sample size given. Kindly clarify.

**DISCUSSION SECTION**

In Page 16 you indicate that the proportion of “matching”. The sentence seems incomplete. Is it matching entries on referral?

In the second paragraph in the discussion section you mention that the reasons for referral do not match the guidelines. In my view – the intention is not to exactly match the guidelines word for word. Whereas the reasons for referral in the RRL book indicate reasons that you mention such as “better patient management” they convey the nuance of the needs of the healthcare worker and should hence not be seen as a radical departure. The guidelines should be seen as guidelines and not an absolute way of filling in a referral form. I suggest that you discuss the evidence on the practical use of the form as a way of describing the departure from the guidelines.

In the fourth paragraph pg. 16 revise spelling for found. The sentence on the limitations also needs to start in its own paragraph.
