# Supplementary material for: Analysis of low resource setting referral pathways to improve coordination and evidence-based services for maternal and child health in Ethiopia
Source: PLoS One. 2022 Aug 25;17(8):e0273436. doi: 10.1371/journal.pone.0273436 (PMC9409580; doi:10.1371/journal.pone.0273436)
Supplement: Attachment — Submitted filename: Reviewer_Report_Plos One_24.11.21.docx [file pone.0273436.s002.docx]

Reviewer’s Report

Title: Analysis of referral pathways in Low-Resource Settings for enabling an evidence-based

point-of-care instrument

**Manuscript ID:** PONE-D-21-20872

**Date:** 24^th^ November, 2021

**Reviewer:** Dorothy Osei

Reviewer’s Report

Thank you for the opportunity to review a manuscript entitled “Analysis of referral pathways in Low-Resource Settings for enabling an evidence-based point-of-care instrument” The study was clear in assessing the referral pathways in Ethiopian Primary Health Care. This is a good study looking at the challenge of feedback in referral pathways in the African context. However, I have a few suggestions that when considered can help improve the manuscript.

Title:

1. The title should include person and time.
2. The title of the article does not fully cover the objective of the research. For instance, there is no point in time in the research findings where the care of patients was analysed using the information on Referral Registration Logbook (RRL) as suggested by the title as an evidence-based point-of-care instrument.

Abstract:

3. From page 8 under the heading Objectives line 1-2. The objective of the study suggest that the analysis is to enable data-driven clinical pathway but the discussion and conclusion of the research largely focused on the completeness and feedback mechanism of the RRL and card-sheet
4. Paragraph 3 of page 8 under the methods portion does not indicate the study type used for the research.

Introduction:

5. From my review the introduction is good.
6. In page 10 line 42-44, the objectives of the research are clearly stated.

Methods:

7. Page 12 paragraph 1 line 3, the study design used for research is quoted as “a case study” but every evidence about how the data was obtained and analyzed indicated that this was a retrospective observational study design.
8. Also, in page 11 paragraph 2, there was no mention of the tool that was used to carry out the study.
9. In page 12 paragraph 3, line 2 – 5 the analysis was overly concentrated on the completeness and feedback of the referrals but did not cover the other objectives of the research.

Results:

10. In page 13 paragraph 3, the study results are well-represented but this does not look at the issues raised in the objectives which include the decision to refer or not to refer a client (to determine unnecessary referrals).
11. In paragraph 3 under results, the issue of timeliness was not duly covered in the table 2: Analysis of RRL data quality metrics
12. Can the results also show information outcome of referrals as a proxy appropriateness of referrals based on the condition for treatment?

Discussion:

13. In page 15, line 3, there should be a space between results, and 11/12.
14. In page 15 line 7, the % sign was omitted from the 72.
15. In page 15 under the discussion, again this does not cover the entirety of the study objectives tough it clearly highlights the bottleneck of referrals as the non-provision of feedbacks from the referral point to the referring facility.

Conclusion:

16. In page 17 paragraph 2 the conclusion indicates that the study addressed the issues of how the referrals decisions were made, the coordination and communication of the referrals but these were not presented in the results and further discussions.
17. In paragraph 2 line 12, the recommendation did not target a specific stakeholder.

References:

Both endnote and in-text referencing need to be worked on in the entire paper. Authors must stick to one referencing style throughout. For instance;

18. In page 9, line 5, amia2011: Authors should consider capitalizing A and provide a space between Amia and 2011
19. In page 9, line 8, Teklu, et al 2020: I suggest the punctuation should come after the et al.
20. In page 9, line 10, Heimly2011: The authors should provide a space between the author’s name and year of publication and a semi-colon after the year of publication.
21. In page 9, line 18, Fehlings & ODonnel: should be stated Fehlings and ODonnel.
22. In page 9, line 20, (Chu, 2001a, Chu, 2001b, DiJerome, 1992 and Donald et al., 2016)): consider the arrangement of the year of publications. I suggest it should start from the oldest to the current year of publication. Also, please be consistent with the typing of the et al, it's either you are using a full stop together with a comma or choosing one of the afore-mentioned punctuation signs throughout. Again, consider closing the in-text reference with just a bracket.
23. In page 9, line 22, Gonz´alez-Ferrer et al. ,2013: Be consistent with formatting, the et al issue again.
24. In page 9, line 24 and 25, (Bettencourt-Silva, Mannu, and Iglesia, 2016; Zhang and Padman, 2017; Bettencourt-Silva, Clark, et al., 2015; Funkner, Yakovlev, and Kovalchuk, 2017; Zhang, Padman, and Patel,2015): Bettencourt-Silva has no year of publication and I suggest the years are arranged from the oldest to the newest.
25. In page 11, line 9 (under Study Setting and Design): (Daniel, W. W., and Cross, C. L., 2018; Israel, G. D., 1992): In-text referencing should be stated appropriately.
