# Supplementary material for: Analysis of low resource setting referral pathways to improve coordination and evidence-based services for maternal and child health in Ethiopia
Source: PLoS One. 2022 Aug 25;17(8):e0273436. doi: 10.1371/journal.pone.0273436 (PMC9409580; doi:10.1371/journal.pone.0273436)
Supplement: Attachment — Submitted filename: Response to Reviewers_v2.docx [file pone.0273436.s004.docx]

**Rebuttal letter**

**Dear Editor,**

PLOS ONE Journal

Website: https://journals.plos.org/plosone/s/journal-information

May 24, 2022

Dear Sir/Madam

Thank you for allowing us the opportunity to submit a revised draft of the manuscript. We thank you and the reviewers for a thorough reading and constructive criticism of our manuscript and for the opportunity to revise and resubmit. We are pleased to submit the improved research article, including a proposed slight title change, “***Analysis of low resource setting referral pathways to improve coordination and evidence-based services for maternal and child health in Ethiopia***” for your consideration in the PLOS ONE. On the following pages, you will find our response to the editor and reviewer comments.

On behalf of my co-authors, I thank you for your consideration of this resubmission. We appreciate your time and look forward to your response.

Please see below, in blue, for a point-by-point response to the reviewers. All page numbers refer to the revised manuscript file with tracked changes. All modifications in the manuscript have been highlighted in red.

Sincerely,

Geletaw Sahle Tegenaw

Reviewer #3: There is a problem with the title. The use of the word' between' suggests that more than one low resource setting was studied, whereas the study was conducted in Ethiopia only.

We appreciate the efforts by you and the reviewers on the manuscript. To better frame the main thesis of our paper, we updated the title to “**Analysis of low resource setting referral pathways to improve coordination and evidence-based services for maternal and child health in Ethiopia**”

Please either use 'low resource setting' or 'poor resource setting’

Thanks for the reviewers comment and we appreciate your suggestion. Now, we updated the 'poor resource setting' to 'low resource setting’.

In the conclusion, you suggest use of automated patient management as a solution- is the infrastructure available? is the human resource trained? what would be the 'cost benefit' implication? Whereas you mention that this is inline with the clinical guidelines, context is very important.

We appreciate the motivation for these comments. We updated and provided context. We added “**when the necessary infrastructure and human resources are in place**”. The whole sentences are as follows:

Therefore, when the necessary infrastructure and human resources are in place, implementing an automated patient management system may help *to correct and impose a standardized referral service workflow*.

Finally, to provide additional explanation and context, we added:

Furthermore, even before viable infrastructure is implemented and competent human resources are attracted, a minimal infrastructure (a low-cost alternative) and trained personnel can facilitate referral coordination and offer evidence-based services to ensure a buy-in and acceptance by all stakeholders. This transition needs careful planning and openness to adopt best practices in order to reduce resource waste, to ensure training, maintenance, and avoid heavy service expenses.
